# ‘A good decision is the one that feels right for me’: Codesign with patients to inform theoretical underpinning of a decision aid website

**DOI:** 10.1111/hex.13844

**Published:** 2023-09-13

**Authors:** Kelly Kohut, Kate Morton, Karen Hurley, Lesley Turner, The CanGene‐CanVar Patient Reference Panel, Caroline Dale, Susan Eastbrook, Rochelle Gold, Kate Henwood, Sonia Patton, Reshma Punjabi, Helen White, Charlene Young, Julie Young, Elizabeth Bancroft, Lily Barnett, Sarah Cable, Gaya Connolly, Beth Coad, Andrea Forman, Helen Hanson, Grace Kavanaugh, Katherine Sahan, Katie Snape, Bethany Torr, Rosalind Way, Elizabeth Winchester, Alice Youngs, The International Lynch Decision Aid Stakeholder Panel, Diana Eccles, Claire Foster

**Affiliations:** ^1^ School of Health Sciences, Centre for Psychosocial Research in Cancer (CentRIC) University of Southampton Southampton UK; ^2^ St George's University Hospitals NHS Foundation Trust London UK; ^3^ Stanford R. Weiss, MD Center for Hereditary Colorectal Neoplasia Cleveland Clinic Cleveland Ohio USA; ^4^ Patient and Public Collaborators; ^5^ Cancer Genetics Unit and Academic Urology Unit The Royal Marsden NHS Foundation Trust London UK; ^6^ Oncogenetics Team The Institute of Cancer Research London UK; ^7^ Division of Genetics and Epidemiology The Institute of Cancer Research London UK; ^8^ Nuffield Department of Population Health, The Ethox Centre University of Oxford Oxford UK; ^9^ Faculty of Medicine University of Southampton Southampton UK

**Keywords:** codesign, coproduction, patient decision aid, shared decision‐making

## Abstract

**Introduction:**

Patient decision aids (PtDA) complement shared decision‐making with healthcare professionals and improve decision quality. However, PtDA often lack theoretical underpinning. We are codesigning a PtDA to help people with increased genetic cancer risks manage choices. The aim of an innovative workshop described here was to engage with the people who will use the PtDA regarding the theoretical underpinning and logic model outlining our hypothesis of how the PtDA would lead to more informed decision‐making.

**Methods:**

Short presentations about psychological and behavioural theories by an expert were interspersed with facilitated, small‐group discussions led by patients. Patients were asked what is important to them when they make health decisions, what theoretical constructs are most meaningful and how this should be applied to codesign of a PtDA. An artist created a visual summary. Notes from patient discussions and the artwork were analysed using reflexive thematic analysis.

**Results:**

The overarching theme was: It's personal. Contextual factors important for decision‐making were varied and changed over time. There was no one ‘best fit’ theory to target support needs in a PtDA, suggesting an inductive, flexible framework approach to programme theory would be most effective. The PtDA logic model was revised based on patient feedback.

**Conclusion:**

Meaningful codesign of PtDA including discussions about the theoretical mechanisms through which they support decision‐making has the potential to lead to improved patient care through understanding the intricately personal nature of health decisions, and tailoring content and format for holistic care.

**Patient Contribution:**

Patients with lived experience were involved in codesign and coproduction of this workshop and analysis as partners and coauthors. Patient discussions were the primary data source. Facilitators provided a semi‐structured guide, but they did not influence the patient discussions or provide clinical advice. The premise of this workshop was to prioritise the importance of patient lived experience: to listen, learn, then reflect together to understand and propose ideas to improve patient care through codesign of a PtDA.

## 1 INTRODUCTION

People who access healthcare (hereafter referred to as ‘patients’ although they may not have any current health concerns) are asked to make difficult decisions, often amidst other competing priorities such as age‐related needs, comorbidities, values, support system and the context of their life situation. Shared decision‐making (SDM) between patients and healthcare professionals (HCPs) is recommended to ensure personalised support to make good decisions^1^, ^2^ and there is a legal obligation to assess capacity^3^ and explain consequences of medical interventions.^4^ However, SDM is challenging to universally implement.^5^, ^6^, ^7^, ^8^, ^9^ Resources are stretched, limiting time in clinic for ‘collaborative deliberation’^10^ and consent‐taking for complex decisions,^11^ although general principles, strategies and evidence communication guidelines can be helpful.^12^, ^13^, ^14^ There are encouraging examples of successful implementation with dedicated funding, training and support from political and healthcare systems,^15^, ^16^, ^17^ resulting in improved satisfaction, cost‐ and time‐savings and redress of the traditional power imbalance in patient‐provider relationships. SDM is worthy of the required effort from patients and clinicians as ‘a method of creating the best care… also the human, kind and caring thing to do’.^18^

Patient decision aids (PtDA) are digital or paper‐based decision support interventions, designed to help with clinical equipoise (where one choice is not necessarily ‘best’), by supporting people to consider decisions in line with their values and priorities.^19^, ^20^ PtDA cannot replace SDM achieved through relationship and consensus building with HCP,^21^ however by incorporating some of the relational processes and mechanisms from behavioural theory, PtDA provide a valuable, patient‐facing resource to support holistic care. A large cochrane review found that PtDA increased knowledge and confidence compared with usual care.^22^ Our research group (K. K., K. M., L. T., C. F., D. E.) conducted a systematic literature review^23^ of decision‐support interventions for genetic testing or care of people with a genetic cancer susceptibility. This identified the potential for these resources to be useful and valued by patients and HCP, however, there was a lack of patient codesign.

Partnering with patients and other experts, our research group is codesigning a PtDA about genetic cancer risk management due to Lynch syndrome, and have described how the accessible template is easily adaptable for other conditions.^24^ An international stakeholder group (see consortium authorship) was established to foster collaboration and successful clinical implementation, to maximise patient benefit. People with higher decision support potentially have the most to gain from PtDA. We are taking a codesign approach with diverse groups of patients, community leaders, artists and low literacy specialists to make the PtDA as accessible and meaningful as possible. This approach has the potential to lead to improved health outcomes^25^ through knowledge mobilisation^26^ and increased access to evidence‐based screening, prevention, diagnosis and treatments.

Theory is important to guide the development of content, format and outcome measurements for PtDA,^27^, ^28^ however use of theory is often omitted or not described,^21^ and there is no one theory considered fit for purpose to support complex decision‐making.

During the first 2 years of PtDA development, we partnered with the CanGene‐CanVar Patient Reference Panel (‘patient panel’) to codevelop content. CGCV is a programme of work funded by the charity Cancer Research UK. Some patient panel members were research partners from conception, while others joined later. We identified that we had not engaged strongly with the patient panel around more theoretical aspects, such as the programme theory through which we hypothesised the decision aid would lead to ‘better’ decisions. Despite developing a logic model which had informed our work, we had not discussed this in depth with the people who would know most about how they make decisions. We discussed this with the patient panel and they were enthusiastic about an idea to hold an interactive workshop to ask what is important to them when they make decisions. The findings would inform the theoretical underpinning and codesign of the PtDA.

### 1.1 Workshop questions

The aims of the workshop were to partner with patients to consider:

1. What constructs from decision‐making theory resonate most strongly or are most relevant and meaningful for patients when they make health decisions?
2. What are the implications for codesign of PtDA, in terms of the underlying theoretical mechanisms of action and potential outcome measures?

## 2 METHODS

### 2.1 Methodological rigour and reporting standards

The Journal Article Reporting Standards^29^ and the Consolidated Criteria for Reporting Qualitative Research checklist^30^ were used to ensure transparency and quality of reporting qualitative data.

### 2.2 Workshop aims

**Figure 1:**
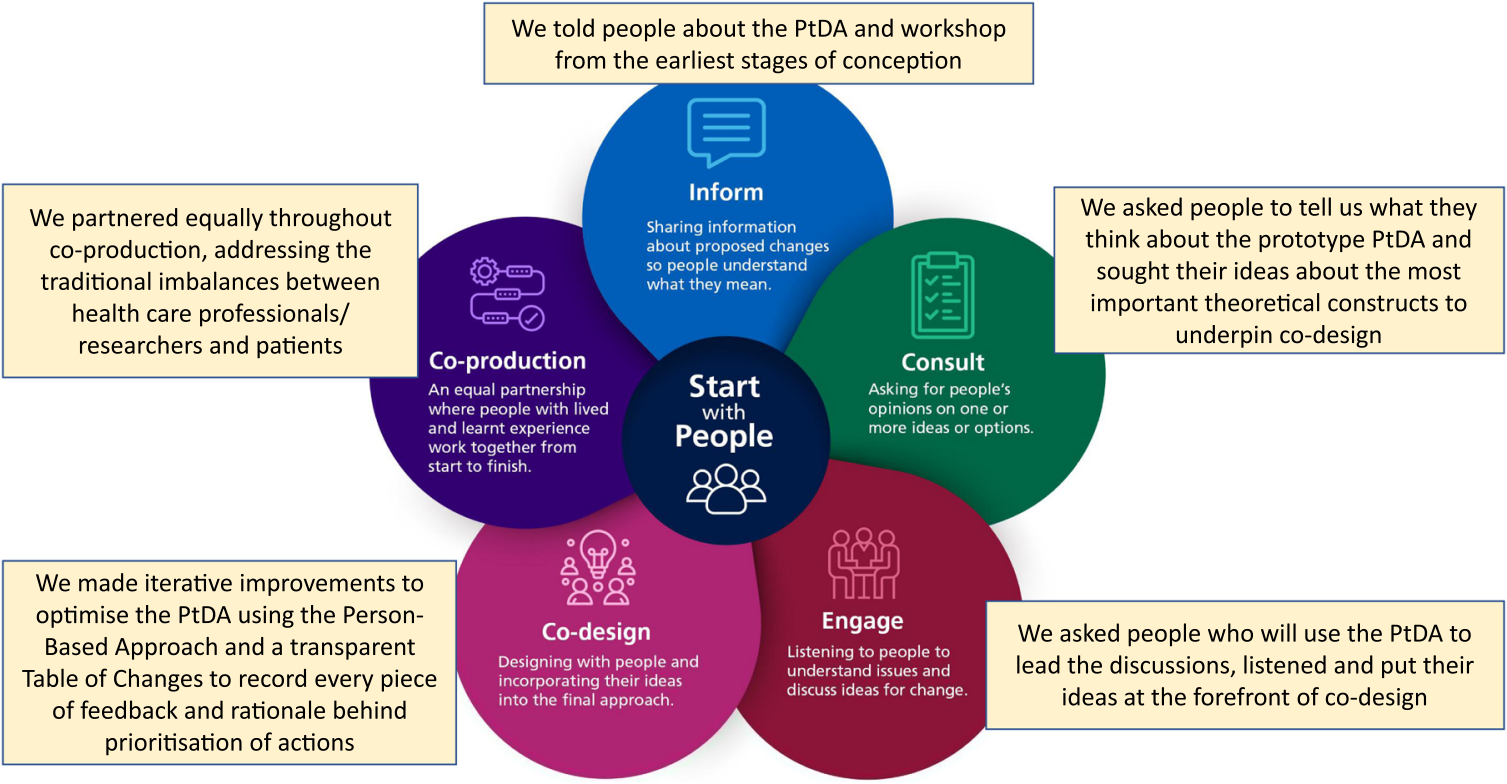
Adapted from NHS England statutory guidance B1762 ‘Working in partnership with people and communities’.^31^ Rectangular boxes show how we followed this guidance when planning and executing the patient workshop and for the codesign and coproduction of the patient decision aid/website. PtDA, patient decision aids.

The aim of the patient workshop was to ensure theoretical underpinning of the PtDA was grounded in what was important to patients with lived experience. Patients were asked to coproduce the workshop and share power and decision‐making before finalising the agenda. We agreed with the patient panel to ‘start with people’^31^ (Figure 1) and ask them to lead discussions considering what is most important to them when they make decisions. We used aspects of coproduction including bringing people together as equal partners, valuing all knowledge, using a creative approach,^26^ keeping in regular contact and setting clear expectations.^25^ These principals were explicitly agreed with patient members before joining the panel, along with the goal of the research programme to codevelop ‘more relevant and acceptable’ and ‘more usable’ products,^26^ in our case a patient website about genetic cancer risk containing values‐based PtDA about decisions such as taking aspirin and risk‐reducing surgery. Learning from patients’ lived experiences would be used to guide codesign of the PtDA for implementation in clinical practice.

### 2.3 Workshop planning

A multidisciplinary planning committee was convened including clinical (K. K., K. H.), research (K. M.), ethics (K. Sa.), management (B. T.) colleagues and the patient acting as chair of the patient panel (L. T.). L. T. was the only patient on the committee due to availability, time and funding constraints but it was made clear her voice and dissemination of the patient panel's views were to be given equal strength and prioritised to drive the direction of workshop planning. L. T. agreed with the rest of the panel in advance that they wished for her to represent them. L. T. communicated with other panel members to gather opinions before decisions were finalised. Starting from conception of the idea for the workshop it was made clear to L. T. and the panel that they would be asked to lead the direction of planning and that the intention was to learn from their lived experiences.

Patients were reimbursed for time spent in meetings or activities, but no professionals received remuneration in addition to their usual paid role as clinicians/researchers. The committee was UK‐based except for K. H. who was invited from the United States due to its international reputation and expertise. The cost of K. H.'s transportation and hotel were covered but she did not receive any other remuneration.

A successful patient workshop^32^ was used as a guide. Guidelines were followed^31^, ^33^, ^34^ to go beyond involvement to build trust, collaboration, quality and impact by sharing power and work towards a shared goal of improving patient care and experience.^35^ The patient panel members all expressed this goal before joining the panel. Feedback from panel members about the experience partnering with researchers to codesign the PtDA has been positive. A paper is planned to highlight this experience in a patient and public involvement journal, to inspire other researchers to form partnerships with patients and take a codesign approach.

K. H. prepared short, simple presentations about theories to inform, orient and sensitise patients about these concepts and initiate discussions about what was important to them.

Facilitators and note‐takers were purposively sampled from local clinical and research cancer genetics services based on expertise and to allow prioritisation of budget for patient expenses. None of the facilitators or note‐takers were patients to allow the patients freedom and comfort to share their lived experiences without having to focus on writing notes, timekeeping or discussion prompts. The facilitators and note‐takers were not paid for their time or expenses, and they were instructed to keep as quiet as possible to listen and learn from the patients. They enthusiastically agreed to this learning opportunity to inform their clinical or research practice.

As a genetic counsellor with >15 years of experience in clinical practice, the lead author (K. K.) was considered an ‘expert’ in a position of power. Although this power dynamic was deliberately addressed by inviting patients to lead discussions and involving them in analysis, it is recognised that K. K.'s existing knowledge and background will have influenced the findings. K. K. acted as a presenter/facilitator and was not directly involved in discussion groups. A preworkshop dinner was offered for organisers and patients. This was designed to provide a relaxed atmosphere to build rapport.

An artist was contracted to create a live visual summary drawing representing a lay interpretation of the concepts discussed. Dissemination of art could be more accessible for those with lower literacy or a preference for visual learning.

### 2.4 Workshop content

**Figure 2:**
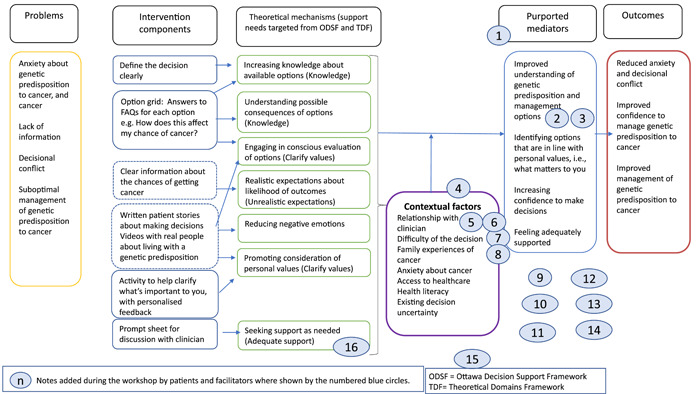
Draft logic model for patient decision aid website/booklet, displayed on the wall in poster size during the workshop. Notes were affixed to this throughout the day with ideas and suggestions from patients and facilitators (shown in circles, numbered). 1. ‘These are our “active ingredients” for decision‐making’. 2. ‘Why doesn't the UK have panel genetic testing?’ 3. ‘Improve equity of access/opportunity for information’. 4. ‘This purple box should move up closer to the beginning’. 5. ‘Clinicians need to remember that we are people too, not just part of the process’. 6. ‘Use of language: clinician + patient ‐ WE’. 7. ‘Is my clinician “on board” with the decision aid usage?’ 8. ‘Heavy decision/burden. What does it all mean? However, clinician often makes this for you’. 9. ‘Having time to consider. Ability to process’. 10. ‘Peer‐peer/patient support groups’. 11. ‘Professional education, especially for rarer disorders’. 12. ‘Cultural/professional stigmas, discrimination concerns’. 13. ‘Empowerment of patient, improved advocacy, specialists’ 14. ‘Urgency: where do we capture this? Treatment versus risk reduction versus age‐related risks’. 15. ‘Timing of decision might be affected by other responsibilities, for example, young children to care for’. 16. ‘Other people's stories: maybe use video testimonials. Less of a “scenario” if it's spoken’.

Table 1 summarises the main theories and theoretical constructs identified as pertinent to underpin PtDA design, including strengths, limitations, and possible adaptations applied to PtDA. The six theories selected for short presentations are highlighted in blue in Table 1 and shown in Figure 2. This is adapted from Elwyn et al.,^27^ who identified eight theories most relevant to decision support interventions through workshops with clinical and academic researchers. The Elwyn paper was used as a starting point given its focus on theory‐based interventions, with more recent papers identified using forward citation‐searching.^56^, ^57^, ^58^, ^59^ A rapid overview of decision‐making theory literature was completed using search teams ‘psychological theory’ or ‘decision‐making theory’ and ‘decision aid’ or ‘decision support intervention’.

**Table 1:** Decision‐making theories organised according to three main types: normative, descriptive and prescriptive.

Type of theory	Examples of theories, theoretical constructs or models	Strengths	Limitations	Applications for codevelopment of PtDA
*Normative*: How should people rationally make decisions, assuming they understand the facts? Used to predict decisions.	Expected Utility Theory.^36^ **Health Belief Model**.^37^, ^38^	Simple. Easy to measure ‘correct’ choice. Elicits perceived risks, severity, benefits, barriers.	Assumes rationality based on ideal conditions. Developed using controlled experiments with limited samples of people. Not designed for decisions with clinical equipoise. Utility may not be synonymous with value for people. Dated.	Decision analytic techniques for example, decision trees, Likert scales, trade‐offs (limited application and evidence).^28^
*Descriptive*: How do people actually make decisions? Used to describe what decisions people make, rather than predict what decisions they should make.	Prospect theory.^39^ Decision framing.^40^ Two types of thinking: fast and slow,^41^ **cognitive heuristics**,^42^ gist/fuzzy trace theory,^43^ **Social Cognitive Theory**,^44^, ^45^ **Self‐Determination Theory**,^46^, ^47^, ^48^ distributed decision making.^49^	Considers how people actively solve problems, use confidence or self‐efficacy. Accounts for emotions and thinking short‐cuts (heuristics). Includes role of social network influences. Decisions may evolve over time involving a range of interactions. Real‐world conditions.	Complex, personal therefore may not be generalisable to other populations. Requires time and effort.	Elicit unobservable thought processes for example, using think‐aloud interviews, questionnaires. Encourage people to slow down, unpack foreclosed decisions and avoid the wrong choice/decision regret. Consider risk communication strategies including simple, visual presentations, reference points and framing. Attention to supporting basic needs: autonomy, competence, relatedness.
*Prescriptive*: How can people be encouraged to make ‘good’ decisions? Aimed to ensure patients understand choices and personal implications so they can decide what is right for them at the time.	Conflict, choice and commitment.^50^ Differentiation and consolidation,^51^ **Rational‐Emotional Model**,^52^ Attend, React, Explain, Adapt model of affective forecasting,^53^ Ottawa Decision Support Framework.^54^	Right or ‘good’ decision is personal, based on understanding the choices and consequences. Addresses cognitive dissonance, impact bias, decision avoidance, fear and uncertainty.	People must desire and accept help with decision making. Trust and rapport essential prerequisites. Requires open mindedness, time, effort and **resilience** to make a deliberative decision in difficult circumstances.	Raise awareness of blind spots, sources of support. Decisional balance sheet. Decisional conflict scale. Personal narratives from people who have made the decision. Promotes concept of ‘informed consent’ (for complex decisions this is increasingly broad consent about the types of results and consequences rather than specifics of every eventuality).^55^

*Note*: Examples of each are given, along with strengths, limitations, and possible applications to codevelopment of PtDA. The six theories chosen for presentation at the patient workshop are highlighted in bold. The theories were chosen based on a rapid overview of the decision‐making theory literature, particularly guided by a paper by Elwyn et al.^27^ as well as planning discussions among the workshop organising team.

Abbreviation: PtDA, patient decision aids.

Planning meetings of the committee with knowledge and experience in psychology, health behaviour, decision support interventions and clinical care along with a patient representative informed the final selection of theories to present. These included two also identified by Elwyn's group (cognitive heuristics and Rational‐Emotional Model) along with four others (Health Belief Model, Social Cognitive Theory, Self‐Determination Theory and resilience/resilient adaptation).

The patient panel Chair (L. T.) asked the panel which theories they thought should be prioritised. The members decided they were happy to let the committee decide. The reason given was because they did not do research before the workshop and came relying on their own lived experience rather than any prior academic knowledge.

The agenda (Table 2) combined short presentations about theoretical constructs designed to educate, stimulate reflection and inspire vibrant group discussions.

**Table 2:** Workshop agenda.

Title: How do people make decisions?	
Time	Content
Sunday evening 9/10/2022	Optional dinner for patients and organisers (provided).
10/10/2022 9:30–10:00	Arrival for the workshop, name badges (refreshments provided).
10:00	Overview of decision aid progress to date and purpose of this morning's discussion *Speakers: K. K. (genetic counsellor), K. M. (health psychologist)* *and K. H. (health psychologist)*.
10:20	Small group discussions about experiences of making decisions.
10:50	Discuss examples from the CanGene–CanVar decision aid.
11:15	Break (refreshments provided).
11:30	Introduction of some ideas from theories about how people make decisions *Speaker: K. H. (health psychologist)*.
12:00	Small group discussions about which are most relevant.
12:30	Feedback to the whole group about key points from discussions.
12:45	Lunch (provided).
13:45	More ideas from theories about how people make decisions *Speaker: K. H. (health psychologist)*.
14:15	Small group discussions about which are most relevant.
14:45	Break (refreshments provided).
15:00	Small group discussions about what the decision aid needs to do to support people, feeding back together at the end.
15:45	Wrap‐up.
16:00	End.

**Figure 3:**
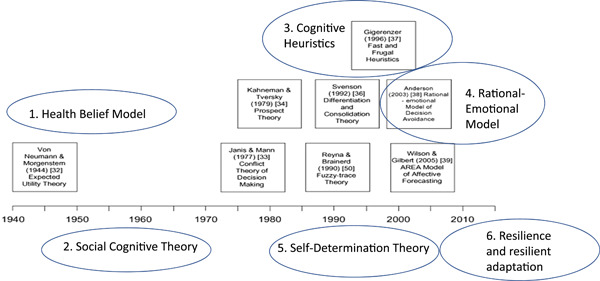
Theories considered and selected for short, lay‐friendly presentations at the workshop. Elwyn et al.^27^ identified eight theories (shown in boxes) published between 1940 and 2010 with the most relevance to design and evaluation of decision‐support interventions. Additional theories or constructs from theories were also considered, based on publications found through forward citation‐searching, a rapid overview of the decision‐making theory literature and discussion amongst workshop organisers with expertise in psychology, health behaviour decision making and clinical genetics. The final theories presented at the workshop are numbered 1–6 and shown in blue ovals.

The draft PtDA logic model (Figure 3) was devised before the workshop, based on theoretical constructs hypothesised to support good decision‐making. Support needs were mainly guided by the Ottawa Decision Support Framework^54^ (based on multiple theories) and the Theoretical Domains Framework^60^ (designed to underpin behavioural interventions). The logic model was displayed on the wall in poster size. Patients and facilitators were invited to affix notes or provide verbal feedback if preferred. It was explained that suggestions would be considered to optimise the logic model.

**Figure 4:**
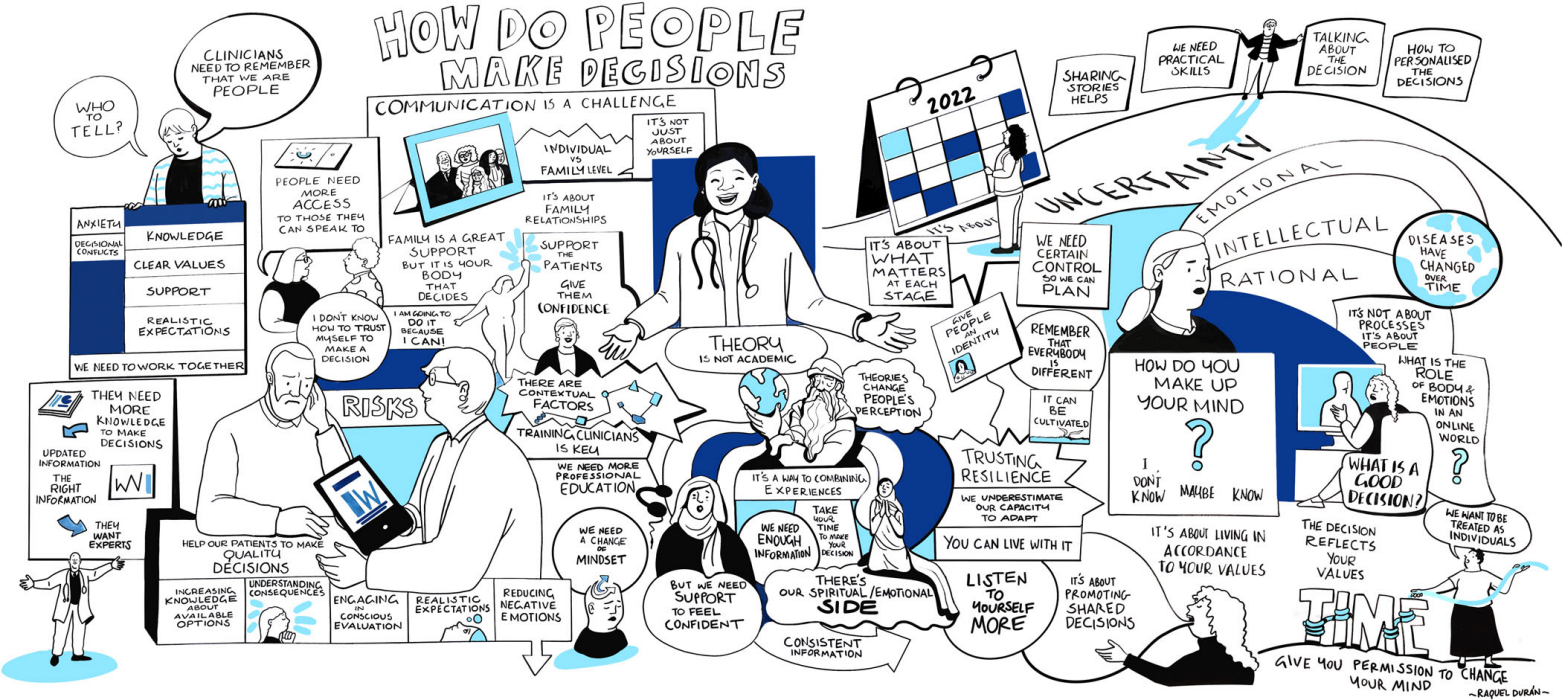
Live visual summary created by a professional artist (Raquel Durán), summarising a lay interpretation of the presentations and the patient experiences and views shared in small group discussions.

The artist's chosen format was a large mural with cartoon illustrations and text, based on prior experience producing live visual summary drawings. The mural (Figure 4) was exhibited on the wall and the artist invited interaction.

### 2.5 Patient recruitment

All 13 members of the patient panel were invited by the Chair (also a patient). To increase diversity and learn from varied perspectives and lived experiences, the manager (B. T.) also contacted the Patient Group lead for BRCA‐DIRECT (a study offering streamlined genetic testing to people with breast cancer), Black in Cancer (an organisation highlighting Black excellence in cancer), the Patient Cancer Research Group for Diverse Backgrounds and South East London Patient Group (reflective of the local community) and The Institute of Cancer Research and The Royal Marsden Patient Engagement Groups (including people who have had genetic testing and/or cancer screening). A trusted leader from each of these groups was asked to share the invitation.

Patient expertise was varied in terms of profession, experience with cancer or as carers and previous involvement in research. Payment for patients' time, dinner and accommodation/travel (if applicable due to living outside London more than an hour's train journey) was provided according to national guidance.^61^ Taxis were arranged if public transportation was difficult due to accessibility issues. Virtual (online) attendance was offered.

The workshop was classified as community engagement rather than research. Therefore, ethics approval was not required. However, techniques were adopted from the research world, with patients agreeing alongside researchers how to analyse and report findings.

### 2.6 Data collection

To ensure comfort sharing personal experiences, a decision was taken with the patient panel not to use audio‐ or videorecording, however at least one facilitator and two note‐takers (genetic counsellors, geneticists or researchers) were present for each small group roundtable or online breakout room discussion. There were more facilitators and note‐takers than patients to enable duplicate notes alongside other tasks such as timekeeping and managing virtual break‐out sessions. These tasks were assigned to facilitators who were all HCPs or researchers, rather than patients. The rationale for this was to allow for patients to have the freedom to focus on sharing their views and lived experiences. Facilitators were instructed to encourage patients to lead discussions, providing minimal prompting because the aim of the workshop was to hear directly from patients what was important to them when they make decisions. Ground rules were agreed, including delineation of tasks and responsibilities and respect for confidentiality and opinions during the discussions. The ground rules had been discussed during the planning committee meetings, including feedback from the patient panel chair on behalf of panel members. The ground rules were presented verbally at the start of the workshop by K. K., and all patients and facilitators were invited to make suggestions either in the group setting or privately. All participants agreed to the ground rules as presented.

Notes were not verbatim, although they included quotes as heard by the facilitators and later checked by the patients. Notes were visible to patients who could ask for corrections. Permission to share photos taken by facilitators on social media was obtained verbally.

### 2.7 Evaluation instruments

Following the workshop, patients, presenters and facilitators were sent a feedback survey.

### 2.8 Analysis

Duplicate notes from each discussion group were compared, combined and analysed using inductive, reflexive thematic analysis.^62^, ^63^ This was chosen as a flexible method applied to qualitative workshop data to answer the questions: What is important to people when they make decisions, and how should this be applied to codesign of a PtDA? Reflexive thematic analysis can ‘reflect reality and… unpick or unravel the surface of “reality”’,^62^ making this a good fit to draw meaning from the discussions.

Notes from discussion groups were reviewed by the lead author (K. K.) to achieve data familiarisation then analysed and interpreted for patterns and grouped into codes and themes descriptively and interpretively. Collaborative coding was achieved by checking quotes, codes and themes directly with patients (all who attended the workshop also agreed to be coauthors). A few quotes were directly corrected by patients using tracked changes of a Word document displaying the quotes organised into themes and subthemes by the lead author. And additional quotes were added to the manuscript following a comment that one patient couldn't locate their ‘voice’ in the selected quotes. A constructionist approach was used to search for meaning together with patients.^64^ This collaborative technique can be used ‘to enhance understanding, interpretation and reflexivity, rather than to reach a consensus’ (p. 8).^63^ All patient coauthors approved the draft and final manuscript before submission.

The draft logic model was revised, applying what was learned from patients to define problems, contextual factors, intervention components, theoretical mechanisms, purported mediators and outcome measures. This was presented to all the patient workshop participant co‐authors for comment and approval before finalisation.

Feedback survey results were analysed with descriptive summaries.

## 3 FINDINGS

### 3.1 Patient characteristics

All invited presenters, organisers, facilitators and note‐takers attended. Roles and titles are listed in Table 3.

**Table 3:** Roles and professional expertise of workshop organisers, presenters, facilitators and note‐takers.

Role for workshop	*n*	Title
Organiser/presenters	1	Genetic Counsellor/PhD student (K. K.)
	2	Health Psychologist (K. M., K. H.)
Project leads	1	Professor of Psychosocial Oncology (C. F.)
	1	Professor of Cancer Genetics (D. E.)
Management	1	Programme Manager (B. T.)
Invitations, room and travel bookings, catering, accommodation, access requirements	1	Research Administrator (R. W.)
Facilitators for small group discussions	2	Consultant Cancer Geneticist (H. H., K. Sn.)
	1	Consultant Research Nurse (E. B.)
	1	Ethics Research Fellow (K. Sa.)
Note‐takers	8	Genetic Counsellor (L. B., B. C., G. C., S. C., A. F., G. K., E. W., A. Y.)
Live visual summary drawing	1	Professional Artist (R. D.)

Ten patients attended. Six were from the patient panel, who all attended in person, including the Chair. One patient from the BRCA‐Direct Group and one from The Institute of Cancer Research Group attended in person. Two from the South East London Group attended virtually, one preplanned and one converted to virtual due to caring responsibilities. Two additional patients from the Cancer Research Group for Diverse Backgrounds who had planned to join virtually were unavailable on the day due to personal circumstances, including caring responsibilities, although funding for carers was offered.

Patient demographics were not collected due to confidentiality. All were female, despite inviting males. Seven out of 10 patients attended the dinner, with most accommodated in a hotel for one or two nights if they lived more than an hour's train journey away.

### 3.2 Thematic analysis of qualitative data

**Figure 5:**
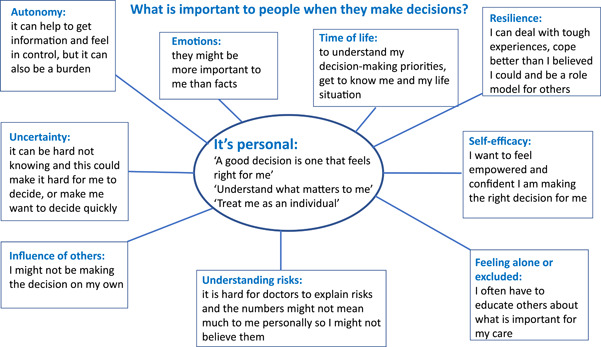
What is important to people when they make decisions? Schematic map of subthemes (shown in boxes): autonomy, emotions, time of life, resilience, self‐efficacy, feeling alone/excluded, understanding risks, influence of others and uncertainty. In the centre (shown in an oval) is the overarching theme: It's personal.

Patient discussions were vibrant, with personal views and lived experiences openly shared. Qualitative data from discussion notes combined with the artist's visual impression were extremely rich. A thematic map is presented in Figure 5, showing the main, overarching theme: *it's personal*. Several subthemes were important to patients when they described how they made decisions: *autonomy, emotions, time of life, resilience, self‐efficacy, feeling alone/excluded, understanding risks, influence of others and uncertainty*. Paraphrased patient quotes were purposively selected to unpack meaning and are presented below.

### 3.3 Overarching theme about how people make decisions: It's personal

Patients had a clear appreciation that decision‐making is highly personal and respected that others might decide differently due to values, personality or life situation:

A good decision is the one that feels right for me.

Your entire life, what you are today is nurture, upbringing. What distinguishes you from me are different experiences. Whether that is cultural, family, descent, which school. Of course, these influence.

Patients expressed an often‐unfulfilled desire for HCP to get to know them and understand their priorities, although they acknowledged institutional and resource pressures that limited time available to invest in this.

Understand what matters to me. Treat me as an individual.

### 3.4 Subthemes

#### 3.4.1 Autonomy

Patients wanted autonomy (the right to decide for themselves). Information helped some to feel in control, although they understood they could not always control outcomes such as their response to medical treatment:

We want control of making decisions. Even though you're not going to stop the inevitable, it feels better to have control.

Preferences for the type and amount of information were often mismatched with what was provided:

How much is too much information you need to make a decision? I wrote my doctor a letter with my pros and cons and what mattered to me, and he came back and thanked me and said he would use it for his patients.

When I was first diagnosed, my oncologist gave me a 50‐page ASCO summary on Herceptin because he knew I wanted information, but that was a bit *too* much.

Autonomy could also feel like a burden, with careful deliberation about complicated medical decisions perhaps too much to bear, especially when dealing with a significant cognitive load from adjusting to a diagnosis and threat to life:

I didn't want the choice. I wanted someone to tell me if I will benefit or not benefit.

Even if they preferred autonomy themselves, patients were respectful that others might prefer to relinquish it when they were unsure what was best, for example about cancer treatment:

Some people will never make a decision for themselves, and they just want to be told. If they want to say, ‘What do I do, doctor?’ that is okay. Sometimes this can validate your decision.

For some people, it's too much. They need to tell someone they trust what's important to them, and then that person can put it together and say what the right path is.

#### 3.4.2 Emotions

An often hidden but vitally important element driving decision‐making was emotions. Patients talked about the personal nature of feelings and life situation, which they recognised that HCP could not necessarily understand.

No one else can feel what you're feeling.

There was a sense that first instincts and ‘going with my gut’ was the right path, no matter how much information was given.

Since my cancer diagnosis, I'm more led by my heart than my mind now.

In clinic, there is a weighting toward factual/rational information. How do we combine the emotional, spiritual, religious with this? For some people, this may outweigh facts.

I don't see mind and emotion as being different. The emotions come from my mind, so they are the same.

Fear was strongly influential, particularly when dealing with shock, information overload and a bewildering choice about complex medical interventions. In the setting of a potentially life‐threatening cancer diagnosis, stakes were high. Fear of death could drive people to rush into a decision hoping for a positive long‐term outcome (survival), even if there were short‐term harms (e.g., side effects from treatment):

I just agreed to anything my doctor said. Because it was life or death.

I just wanted to live. I didn't necessarily think about how living would be, but I just wanted to live.

#### 3.4.3 Time of life

Priorities such as time of life and context were crucially important to personalised decision‐making, but often not shared with HCP if time, support and interest were felt to be lacking.

Thinking back, my decision was based on my kids being young and reconstruction surgery could all be done at the same time.

I was in a long‐term relationship, etc, [not having reconstruction] wasn't a difficult decision as it didn't feel important, and it would have been a big, multi‐step surgery.

New job, no sick leave. I made the decision practically, wanted to limit time off after surgery.

I worry about my kids as they are making decisions at a different time in life. I was married and had my kids.

#### 3.4.4 Resilience

Capacity for resilience surprised people when faced with challenging circumstances. They inadvertently became role models and an inspiration for others. They did so because they needed to get through and find inner strength. They were quietly proud of their resilience, did not always find it particularly helpful for others to comment on it and sometimes found excessive concern unhelpful:

I had to stay positive for my family. It's hard for onlookers, easier for us going through it.

I hate it when people say you are brave. What choice do you have?

Yes, you become resilient. You can't live your life in a ‘river of cream’. You get bad experiences, but if you have responsibilities on your shoulder, you learn to cope.

I'm amazed at how resilient I have become. Took me two or three years afterwards to realise—it's said, ‘You are so brave’ but for me it was contextual factors—family, children…

#### 3.4.5 Self‐efficacy

Self‐efficacy was achieved through belief in ability to understand information and make good choices. When faced with a medical decision, people often drew on prior professional training and experience and applied these skills:

I look at the facts and I make a decision. I was trained that if you find out a decision is wrong then you are not afraid to change your mind. It is not a sign of weakness. Because of that training you don't regret the decisions you make.

I ran my own company—knew I had made bad decisions in past, but felt I'd never looked back and had always learned from these. Regret is an okay thing in life.

Sometimes people needed help finding their inner self‐efficacy, especially during a turbulent time. Support needs were diverse.

There are lots of different ways of giving people help to move forward. Helping people get unstuck: oiling the lock.

Patients often coped better than they thought they would with difficult experiences, showing confidence to make the right decision for them at the time, and kindness to accept that they did their best, which minimised decisional regret later:

For me and the decision I made for my son—I felt confident. It had to be right for me.

#### 3.4.6 Feeling alone/excluded

Patients could feel alone when dealing with a diagnosis, and exclude themselves from accessing potential sources of support:

There is discrimination.

I didn't tell anyone at work.

When deciding against medical advice, this could be a lonely experience and they had to be true to personal values and choices:

How do you trust yourself when a clinician is telling you something else? I know how I feel. I know what I'm about. And I'm in control of this.

It all comes down to your inner voice. You have no choice but to survive and if you don't like the sound of their plan, don't be afraid to say what you want. You know your body best.

People navigating care after a diagnosis felt isolated when their HCP was not well‐informed, which was frustrating and placed pressure on them to be proactive and advocate for themselves:

First stumbling block is education of healthcare professionals… Patients are often more educated than professionals.

#### 3.4.7 Understanding risks

Understanding risks was perceived to alleviate the anxiety and decision paralysis caused by uncertainty:

Knowing about the genetic risk has been key to it all.

However, it was acknowledged that effective risk communication is incredibly challenging, especially in a time‐pressured environment:

Doctors don't even understand absolute and relative risk, which is irritating. How is a patient supposed to?

Treatment or no treatment? Should I, or shouldn't I? I understood the biology, but it was hard to see what the benefits were versus the harms.

Personalised risk communication was important, including age‐stratified risks and considering people's past experiences of medical conditions and treatments and how this influenced perspectives and decision‐making:

The information that is important is different for different people.

Illness from treatment meant menopause didn't feel so big.

#### 3.4.8 Influence of others

External influence was often strong, including from HCP, family, friends and peers. Patients highlighted that they were not making decisions on their own:

The medics need to remember we are people, we have other people, we have families and other people involved in the decision. It's not just the patient in front of you.

My husband was not keen on me having chemotherapy and the pressure of coping with a partner having chemotherapy. This did not sway my decision, but it was a factor. A chat with a pathologist friend helped as she knew the science but was also familiar with my values.

Conflicting opinions and influence could cause pressure and a crisis of confidence for patients already dealing with heightened distress:

You have to make the decision, but everyone around you has their opinion. Some valuable, some not. All of that is sitting on you while you are feeling overwhelmed. You have to quickly make decisions, put your trust in either them or yourself, and hope you're both on the same page, that what they feel is best for you, you feel is right for you. And then you hope you have made the right decision.

#### 3.4.9 Uncertainty

Tolerance for uncertainty was varied. Not knowing could be hard, which could encourage a quick decision to avoid deliberation when cognitive load was high. However, patients showed acceptance that they did the best they could.

The ‘maybe’ phase can be very long—some find it more distressing making a decision whereas others prefer to make a decision and move on.

I only felt anxious while making the decision—once made, I could park it and not regret it. Now it feels like the right decision, in hindsight.

Career was my top priority, and then all of a sudden it was health. Everything shuffled around. Your life feels pretty certain, and then you're in a world of uncertainty.

### 3.5 Artwork

The artist's lay impression (Figure 4) of the messages conveyed by the presenter and patients showcased similar sub‐themes to the thematic analysis of discussion notes:

Clinicians need to remember that we are people.

The potential for the PtDA to complement personalised counselling was highlighted in the artwork:

Help our patients to make quality decisions.

It's about promoting shared decisions.

Support the patients. Give them confidence.

A blog post was shared by the PRP Chair (L. T.) on the research programme website including the artwork and photos: https://www.cangene-canvaruk.org/post/patient-decision-making-workshop. A digital image of the mural will be included on the PtDA website.

### 3.6 Logic model

**Figure 6:**
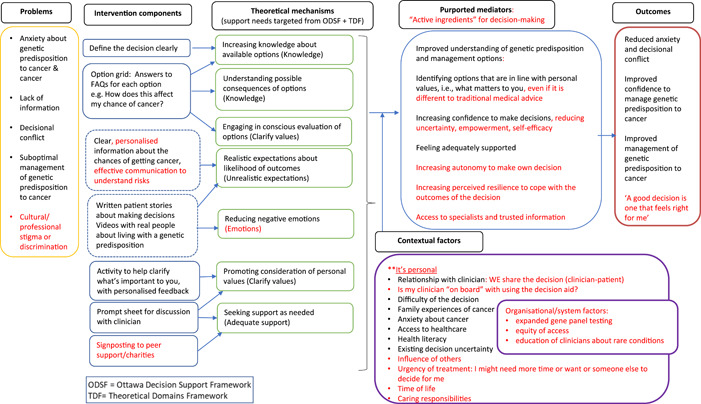
Revised logic model based on output from the workshop, including thematic analysis of patient small group discussions and artwork summary drawing and notes placed on the logic model poster.

Notes placed on the logic model poster (displayed in Figure 2) echoed key considerations raised in patient discussions. The revised logic model is presented in Figure 6, with additions based on patient input shown in red.

### 3.7 Evaluation surveys

#### 3.7.1 Patient evaluation survey

Seven out of 10 patients responded. Descriptive summaries are presented in Table 4. All respondents rated their overall experience as ‘very good’.

**Table 4:** Summary of patient and public collaborator evaluation survey responses.

Survey question	Rating	*n* (%)
1. How would you rate your overall experience of being involved in the decision‐making workshop?	Very good	7 (100)
2. How much do you agree with the following statements?		
(a) The aims of the workshop were clear.	Strongly agree	6
	Agree	1
(b) The workshop was interactive and I had opportunity to contribute.	Strongly agree	7 (100)
(c) I believe my participation has been valuable and will make a difference to the research.	Strongly agree	3
	Agree	4
(d) Any comments about the above?	‘Listening to ideas and opinions of others contributes to the fund of knowledge’.	
	‘Would like feedback from facilitators on how the information from our personal stories will impact on their research’.	
3. What do you think worked well?	‘Short sessions and working with different people’. ‘The artistic representation was very powerful and a fantastic snapshot’. ‘All was perfect. The technology was very well organised’. ‘Small groups of patients’. ‘The agenda was slick, timely, and never felt laboured or rushed. Great example of PATIENT PARTICIPATION done well with parity of participation’.	
4. Is there anything you would like to see done differently going forwards?	‘Feedback given after each session’. ‘Patients changing groups after every discussion with different facilitators’. ‘Maybe consider copresentation with the Patient Reference Panel?’ ‘When we went into breakout groups it wasn't always entirely clear what we were being asked to do, and the facilitators weren't always sure either!’	
5. Please can you let us know if you came in person or attended online?	In person Online	5 2
6. How long do you think our next workshop should be?	2 Days 1 Day Half‐day	1 5 1

*Note*: Rating scales included: very good, good, poor, very poor or strongly agree, agree, disagree, strongly disagree. Only ratings that had at least one response have been shown.

#### 3.7.2 Facilitator/note‐taker/presenter evaluation survey

Thirteen out of 15 responded. Descriptive summaries are presented in Table 5. Facilitators were impacted by patient stories and reflected on application to clinical practice:

Reaffirmed there is not a ‘one ‐size fits all’ for decision making.

**Table 5:** Summary of responses from organiser, facilitator and note‐taker evaluation survey.

What do you think was done well?	What could have been better?	How will what you learned on the day change your thinking? Will you bring any change back to your clinical practice or research?	Please add any other comments
The whole event was very inclusive.	We really needed 2 days as there was so much to talk about.	It has made me think about how individuals make decisions and not to assume.	Extremely well run. Everyone had the opportunity to be heard.
Enjoyed flow of lecture followed by break outs to discuss topics in more personalised detail.	It was important to have someone facilitating online as well as having a body in the room.	Good reminder of how different patient experiences can be. It reminded me not to generalise!	Probably the best organised and useful session I have attended. I also really enjoyed the artist!
Good organisation, loved mixture of lectures, discussions and artwork.	Can't think of anything!	Important to hear patient voice and think about how to facilitate shared decision‐making.	
Running of the day incredibly smooth. Excellent to have regular points for discussion. Having an artist was fantastic.	It may have been helpful to have seen more of the decision aid and the website to help in the discussion.	Shared decision‐making was a key theme. Some make decisions for family rather than on an individual level.	Incredibly grateful to patients for sharing stories, giving a unique perspective of journey in healthcare.
The setting was really interactive. The artist was also a particularly brilliant addition.	It may have helped to have designated hybrid facilitators.	Consider similar for future research and the use of artists in sci comms more.	
Splitting into chunks helped engagement and kept momentum	Hybrid links (but recognise difficulty with this).	Reaffirmed there is not a ‘one ‐size fits all’ for decision making.	Very enjoyable and productive day
Well organised, technology mostly worked. Impressive to include those who could not travel in.	More time for discussion, but that was not possible within the day.	Reminded how nice it is to have face‐to‐face interactions, and the quality of those interactions.	It was so interesting hearing the patients' stories.
Conversations were facilitated but not rigidly, allowing for the richness of true and personal reflections to emerge.	More time walking through the decision aid as a group. Trying to recruit more male patients.	Already changed the way I think—greater confidence in my/my patient's need to make health decisions for themselves.	
It was so well organised, and people were rotated around.	Maybe an ice breaker exercise at the beginning.	Everyone listened to with respect and patience. Every voice counts no matter how big or small.	I felt so honoured to be able to help out.
Very well organised and gave lots of opportunities for people to speak, including virtual patients.	Rotating groups between tables got a bit haphazard towards the end!	Reiterated that there is a huge variety in how people make decisions. Thinking about this more in clinic.	
Strong focus on patient voice. Diverse and engaging content. Respectful atmosphere.	More directed prompts at the beginning of each roundtable would have been helpful.	Individuality—what was preferable to one patient was unhelpful, or even off‐putting, to another.	Overall, a wonderful and informative experience!
Preworkshop meal valuable to build rapport. Visual summary an interesting idea.	Possibly in future increasing the ratio of patients to note takers/facilitators.		

They were impressed with content and inclusion of the artist but suggested a longer workshop and more assistance with facilitator instructions and virtual break‐out rooms.

## 4 DISCUSSION

Our innovative patient workshop produced rich qualitative data suggesting the way people make health decisions is intricately personal. Contextual factors were key and changed over time. Patients were often influenced by others, which in addition to their HCP could include family, friends and peers. They felt obliged to make complex, difficult choices under pressure, due to urgency of treatment, strength of medical advice, or the desire to ‘get on’ and alleviate uncertainty and distress. However, the potential for decision regret was a concern. It took empowerment, confidence and self‐kindness to accept that a decision was the best one at the time, even if they might have made a different decision with the benefit of hindsight.

Simple presentations about theoretical constructs sparked vibrant discussions in an open environment with sharing of personal opinions, including many that were withheld from traditional encounters with HCP during treatment planning. Patients were interested in concepts from many theories but did not feel that the way they make decisions was explained by any one theory. Patients highlighted the importance of contextual factors, suggesting these are underrepresented in traditional decision‐making theories.

We adopted a codesign approach by giving patients who will use health resources a voice and using a creative workshop to develop a shared understanding and find meaning together.^26^, ^31^, ^35^ In line with previous literature,^27^, ^28^ our findings suggest that there is no one ‘best fit’ theory to explain what is important to people when they make decisions, and to underpin a PtDA. We will use an inductive approach to our programme theory, incorporating constructs from multiple theories based on what patients identified was important to them.

Many of the concepts raised by patients as important to medical decision‐making agree with those reported in qualitative studies of people with Lynch syndrome^65^, ^66^ and other genetic cancer susceptibilities.^67^, ^68^, ^69^ These included including challenges with risk communication, frustration about lack of access to expert practitioners, personalised preferences for the amount and format of information, varied personal meaning taken from risk assessments and implications for consideration of management choices. Listening to people's lived experiences in the context of real‐life situations and competing priorities provided novel insight into perspectives and worldviews and presents a unique contribution to the literature.

Our findings add value to those from previously reported studies by asking a group of patients with lived experiences to consider what is important to them as they make decisions generally in life as well as specifically with respect to medical care. Future work is ongoing to partner with more people from diverse groups in the community so we can ensure that the PtDA is helpful and engaging for them.

### 4.1 Strengths and limitations

A major strength of this workshop was prioritising patients' lived experiences, views and preferences as the most important factors to determine incorporation of constructs from decision‐making theories to underpin the conceptual framework of a PtDA. Only one patient (chair of the patient panel) was included on the planning committee due to constraints on funding, time and availability, however panel members were asked to contribute their thoughts, opinions and suggestions before any final decisions were made. Invitations were extended to other research, community and charity patient groups to increase equality, diversity and inclusion. Attendance at the workshop was challenged by a low response rate to these invitations as well as limitations on the size of the room and available funding to cover travel, meals and reimbursement for patients' time. Additionally, even patients who were interested to attend experienced personal circumstances such as health issues or caring responsibilities that meant they had to cancel at short notice or convert planned in‐person attendance to virtual.

Discussions were not recorded to encourage open sharing; therefore, thematic analysis was completed on notes rather than verbatim transcripts which may have resulted in some comments being missed or misinterpreted. To minimise this, two note‐takers were allocated to each group and notes were checked by the patients, all coauthors. The inclusion of a professional artist sketching a live, visual summary provided a vibrancy to the workshop and the final image presented another perspective to showcase the findings and complement the discussion notes.

Although men were invited, none attended, therefore we were unable to explore the views of men or people with other gender identities. Strategies to include diverse patient and researcher partners from underserved groups are being pursued.

## 5 CONCLUSIONS

This paper makes a unique contribution to knowledge, providing rich insight into patient decision‐making priorities, experience and outcomes, as well as giving a worked example of a theory‐focused patient engagement event.

The major finding was that decision‐making is highly personal. There was no one theory or construct to adequately target the range and breadth of support needs for patients making health decisions. Learning from patient experiences led to revised conceptual and logic models for the PtDA. These are being continually refined and optimised using the person‐based approach.^70^ We recommend strategies for patient codesign of PtDA, asking people who will use these resources to partner with researchers to address challenges and solutions together.

## 6 FUTURE DIRECTIONS

Feedback from HCP suggested impactful learning taken back to clinical and research practice. As one summarised, the experience reinforced the importance of personalised SDM: ‘Good reminder of how different patient experiences can be. It reminded me not to generalise!’ Our research group has progressed plans to disseminate and champion our codesign approach and inspire others to consider similar methods. Further codesign workshops are planned, focussing on key topics identified by patients, such as living with uncertainty.^71^, ^72^

Additional, dedicated funding has been secured to increase equality, diversity and inclusion, collaborating with trusted leaders, patients, carers, charities and peer groups. Cultural tailoring and translation of the PtDA will increase accessibility. Longitudinal studies are needed to determine whether use of PtDA leads to improved health outcomes^26^ as part of personalised, holistic healthcare realised in respectful partnerships with HCP committed to SDM.

## AUTHOR CONTRIBUTIONS

**Kelly Kohut**: Conceptualisation; data curation; formal analysis; investigation; methodology; project administration; visualisation; writing—original draft, writing—review and editing. **Kate Morton, Claire Foster, Diana Eccles**: Supervision; methodology; writing—review and editing. **Karen Hurley**: Methodology, writing—review and editing. **Lesley Turner, Caroline Dale, Susan Eastbrook, Rochelle Gold, Kate Henwood, Sonia Patton, Reshma Punjabi, Helen White, Charlene Young, Julie Young, Elizabeth Bancroft, Lily Barnett, Sarah Cable, Gaya Connolly, Beth Coad, Andrea Forman, Helen Hanson, Grace Kavanaugh, Katherine Sahan, Katie Snape, Elizabeth Winchester, Alice Youngs**: Methodology, writing—review and editing. **Bethany Torr, Rosalind Way**: Project administration; methodology; writing—review and editing. **CanGene‐CanVar Patient Reference Panel Members**: Writing—review and editing. **The International Lynch Decision Aid Stakeholder Panel Members**: Writing—review and editing.

## CanGene‐CanVar PATIENT REFERENCE PANEL MEMBERS

Caroline Dale, Sue Duncombe, Rochelle Gold, Sonia Patton, Warren Rook, Richard Stevens, Lesley Turner, Frankie Vale, Helen White, Ivan Woodward, Steve Worrall, Julie Young.

## THE INTERNATIONAL LYNCH DECISION AID STAKEHOLDER PANEL MEMBERS

Munaza Ahmed, Lyndsy Ambler, Antonis Antoniou, Stephanie Archer, Ruth Armstrong, Elizabeth Bancroft, Kristine Barlow‐Stewart, Lily Barnett, Marion Bartlett, Julian Barwell, Dany Bell, Cheryl Berlin, Matilda Bradford, John Burn, Sarah Cable, Dharmisha Chauhan, Ruth Cleaver, Beth Coad, Gayatri Connolly, Gillian Crawford, Emma Crosbie, Victoria Cuthill, Tabib Dahir, Karina Dahl Steffensen, Eleanor Davies, Glyn Elwyn, Mary Jane Esplen, D. Gareth Evans, Pia Fabricius, Andrea Forman, Kaisa Fritzell, Claire Giffney, Joana Gomes, Elizabeth Half, Rebecca Hall, Helen Hanson, Menna Hawkins, Deborah Holliday, Roberta Horgan, Karen Hurley, Margaret James, Ros Jewell, Sarah John, Siobhan John, Victoria Kiesel, Anna Koziel, Anjana Kulkarni, Fiona Lalloo, Helen Liggett, Aela Limbu, Kate Lippiett, Anne Lowry, Manami Matsukawa, Tracie Miles, Shakira Milton, Pål Møller, Kevin Monahan, Laura Monje‐Garcia, Gabriela Moslein, Alex Murray, Jennie Murray, Kai‐Ren Ong, Anbu Paramasivam, Chris Patch, Alison Pope, Sarah Pugh, Imran Rafi, Gabriel Recchia, Nicola Reents, Peter Risby, Neil Ryan, Sibel Saya, Raza Sayyed, Salma Shickh, Toni Seppala, Lucy Side, Sian Smith, Tracy Smith, Tristan Snowsill, Dawn Stacey, Eriko Takamine, Katrina Tatton‐Brown, Helle Vendel Petersen, Robert Volk, Jennifer Wiggins, Lisa Wilde, Jennet Williams, Catherine Willis, Elizabeth Winchester, Kristi Withington, Emma Woodward, Alice Youngs.

## CONFLICT OF INTEREST STATEMENT

The authors declare no conflict of interest.

## ETHICS STATEMENT

All patients consented to being named coauthors and to having paraphrased quotes presented, without personal identifiers.

## Data Availability

The anonymised data that support the findings of this study are available on request from the corresponding author. The data are not publicly available due to privacy restrictions for the patient contributors.
